# Emerging SARS-CoV-2 Variants in Uganda in the Era of COVID-19 Vaccination

**DOI:** 10.3390/v16121860

**Published:** 2024-11-29

**Authors:** Nicholas Bbosa, Ronald Kiiza, Alfred Ssekagiri, Hamidah Suubi Namagembe, Stella Esther Nabirye, Danstan Kabuuka, Cleophous Rwankindo, Annet Kisakye, Yonas T. Woldemariam, Sylvia Kusemererwa, Terry A. Ongaria, Ayoub Kakande, Andrew Abaasa, Geofrey Kimbugwe, Henry Kyobe Bosa, Alfred Driwale, Jason M. Mwenda, Archibald K. Worwui, James Humphreys, Sandra Cohuet, Alison M. Elliott, Eugene Ruzagira, Pontiano Kaleebu, Deogratius Ssemwanga

**Affiliations:** 1 MRC/UVRI & LSHTM Uganda Research Unit, Entebbe 256, Uganda; ronald.kiiza@mrcuganda.org (R.K.); hamidah.namagembe@mrcuganda.org (H.S.N.); sylvia.kusemererwa@mrcuganda.org (S.K.); terry.ongaria@mrcuganda.org (T.A.O.); ayoub.kakande@mrcuganda.org (A.K.); andrew.abaasa@mrcuganda.org (A.A.); geofrey.kimbugwe@mrcuganda.org (G.K.); alison.elliott@lshtm.ac.uk (A.M.E.); eugene.ruzagira@mrcuganda.org (E.R.); pontiano.kaleebu@mrcuganda.org (P.K.); deogratius.ssemwanga@mrcuganda.org (D.S.); 2 Uganda Virus Research Institute, Entebbe 256, Uganda; assekagiri@gmail.com (A.S.); snabirye@uvri.go.ug (S.E.N.); danstankabuuka@gmail.com (D.K.); mwinekansiime93@gmail.com (C.R.); 3 Department of Infection Biology, Faculty of Infectious and Tropical Diseases, London School of Hygiene & Tropical Medicine, London WC1E 7HT, UK; 4 World Health Organization (WHO) Country Office, Kampala 256, Uganda; kisakyean@who.int (A.K.); TegegnYonas@outlook.com (Y.T.W.); 5 Department of Infectious Diseases Epidemiology, London School of Hygiene & Tropical Medicine, London WC1E 7HT, UK; 6 Ministry of Health, Kampala 256, Uganda; hskyobe@gmail.com (H.K.B.); driwalealfred2019@gmail.com (A.D.); 7 The African Region Monitoring Vaccine Effectiveness (AFRO-MoVE) Network, World Health Organization—Regional Office for Africa, Brazzaville 99324, Congo; jmmathiu@yahoo.co.uk (J.M.M.); worwuia@who.int (A.K.W.); 8 Epiconcept Company, 75011 Paris, France; j.humphreys@epiconcept.fr (J.H.); s.cohuet@epiconcept.fr (S.C.); 9 Clinical Research Department, London School of Hygiene & Tropical Medicine, London WC1E 7HT, UK

**Keywords:** SARS-CoV-2, COVID-19, variants, omicron, vaccine, next-generation sequencing

## Abstract

The emergence of SARS-CoV-2 variants has heightened concerns about vaccine efficacy, posing challenges in controlling the spread of COVID-19. As part of the COVID-19 Vaccine Effectiveness and Variants (COVVAR) study in Uganda, this study aimed to genotype and characterize SARS-CoV-2 variants in patients with COVID-19-like symptoms who tested positive on a real-time PCR. Amplicon deep sequencing was performed on 163 oropharyngeal/nasopharyngeal swabs collected from symptomatic patients. Genome assembly, lineage classification and phylogenetic analysis was performed using the Edge Bioinformatics pipeline version 2.4.0, Pangolin version 4.3.1 and iqtree version 2.3.6 software respectively. Of the 163 deep sequences analyzed between April 2023 and March 2024, the most common were XBB.1 lineages and sublineages (113, 69.3%), followed by JN.1* (12, 7.4%), XBB.2* (11, 6.7%) and FL* (11, 6.7%), EG* (7, 4.3%), others (BQ.1.1, FY.4.1, FY.4.1.2, GY.2.1, HK.27.1) (5, 3.1%) and CM* (4, 2.5%). XBB.1* dominated from April to July 2023; thereafter, other variants, including JN.1* were increasingly detected. There was no statistically significant association between vaccine status and lineage assignment (Fisher’s exact test, *p*-value = 0.994). Our findings showed that the Omicron variant, specifically the XBB.1* lineage, was the dominant circulating virus. However, the emergence of the JN.1 variant that exhibits a significant spike protein mutation profile could impact COVID-19 transmission in Uganda.

## 1. Introduction

The Medical Research Council (MRC)/Uganda Virus Research Institute (UVRI) & London School of Hygiene and Tropical Medicine (LSHTM) Uganda Research Unit has contributed toward Uganda’s SARS-CoV-2 response since the beginning of the coronavirus disease 2019 (COVID-19) pandemic [1,2,3]. Uganda is in its fourth COVID-19 wave with a declining number of positive cases. During the first wave that occurred between December 2020 and January 2021, the A.23.1 variant dominated [2]. The second wave occurred between May and July 2021, when the Delta variant was the dominant circulating virus. The third wave began around December 2021 and was largely driven by both Delta and Omicron at the beginning [3] until Omicron replaced Delta to become the dominant variant to date. The SARS-CoV-2 Omicron variant (B.1.1.529) evolved with 10 genetic mutations within the viral receptor-binding domain and at least 32 genetic alterations in the spike protein and was classified as a Variant of Concern (VoC) in November 2021 by the World Health Organization (WHO) [4]. The Omicron JN.1 (BA.2.86.1.1) lineage emerged around August 2023 after evolving from the BA.2.86 commonly known as “Pirola” and quickly became the most prevalent lineage in numerous countries that include the USA, China, Singapore, and India [5]. JN.1 harbors a hallmark Leu455Ser spike protein mutation as well as three mutations in non-spike proteins, and compared to other lineages in the same group, it is more immune system evading and more easily transmissible [6]. There is currently no evidence to prove that JN.1 causes more severe illness relative to other lineages but it has been reported to spread more easily and cause mild to moderate illness [5]. 

Multiple VoCs and variants of interest (VoIs) have been designated by the WHO based on their potential for expansion and replacement of prior variants and for causing new waves that are associated with increased circulation or illness [7]. The advent of the alpha, beta, and delta SARS-CoV-2 variants was linked to new waves of infections globally. The virus continues to evolve as a result of genetic mutations or recombination in the viral genome during replication. The Omicron variant accounts for over 98% of the publicly available sequences since February 2022 and constitutes the genetic background from which new SARS-CoV-2 variants will likely emerge [8].

Since the global rollout of COVID-19 vaccines, there has been remarkable progress in reducing the burden of the disease [9,10]. To date, six vaccines have been approved for use in Uganda that include, Jcovden (Johnson & Johnson Innovative Medicine, Beerse, Belgium), Vaxzevria (Oxford University/AstraZeneca, Oxford/Cambridge, UK), Comirnaty (Pfizer/BioNTech, Mainz, Germany), Spikevax (Moderna, Cambridge, MA, USA), Sinopharm (Beijing Institute of Biological Products, Beijing, China) and CoronaVac (Sinovac Biotech, Beijing, China) [11]. The national COVID-19 vaccine uptake and coverage reported between March 2021 and June 2022 was 63% for ≥1 dose and 42% for ≥2 doses, respectively [12]. However, the emergence of SARS-CoV-2 variants that are capable of evading vaccine-induced immunity poses new challenges to public health efforts globally [13,14]. The purpose of this study was to characterize SARS-CoV-2 variants in patients who participated in a COVID-19 Vaccine Effectiveness and Variants (COVVAR) study at health facilities in Uganda. The study aimed to investigate the prevalence and mutation profiles of the different variant types and explore any associations between vaccination status and these variants.

## 2. Materials and Methods

### 2.1. Study Design and Population 

The COVVAR study is a hospital-based test-negative case–control study with the objective of estimating the effectiveness of licensed COVID-19 vaccines against laboratory-confirmed COVID-19 disease. The study was conducted by the MRC/UVRI and LSHTM Uganda Research Unit at selected health facilities in Kampala, Greater Masaka (Bukomansimbi, Kalungi, Kyotera, Lwengo, Lyantonde, Masaka, Rakai, Sembabule) and Wakiso districts. The study population included vaccine-eligible patients aged ≥12 years who presented with COVID-19-like symptoms.

### 2.2. Case Definition and Recruitment

#### 2.2.1. Case Definition

Any vaccine-eligible patients who presented to a designated health facility with symptoms suggestive of COVID-19 and subsequently had a positive SARS-CoV-2 real-time PCR test.

#### 2.2.2. Control Definition

Vaccine-eligible patients who presented to a designated hospital with symptoms suggestive of COVID-19 and subsequently had a negative SARS-CoV-2 real-time PCR test.

#### 2.2.3. Recruitment

Participants were recruited from outpatient clinics, emergency departments and wards of participating health facilities. A total of 1483 (55.3% female) patients with COVID-19 symptoms were screened. Of those screened, 1398 (94.3%) were enrolled between March 2023 to March 2024. The WHO case definition for identifying COVID-19-like symptoms within the last 10 days was used [15]. This included persons who presented with acute onset of fever and cough or acute onset of any three or more of the following symptoms: fever, cough, general weakness/fatigue, headache, myalgia, sore throat, coryza, dyspnoea, anorexia, nausea, vomiting, diarrhea or altered mental status. Reasons for exclusion included having experienced symptoms for a period that exceeded more than 10 days (83, 5.6%) and being <12 years of age (2, 0.1%). 

#### 2.2.4. Vaccination Status 

Of those enrolled, 802 (57.4%) were fully vaccinated and 52 (6.5%) had received a booster vaccine dose. About the type of vaccine received, 492 (35.2%) received the Vaxzevria (Oxford University/AstraZeneca) vaccine, 288 (20.6%) received the Comirnaty (Pfizer/BioNTech) vaccine, 103 (7.4%) received the Jcovden (Johnson & Johnson) vaccine, 33 (2.4%) received the Spikevax (Moderna) vaccine and 15 (1.1%) received the Sinopharm (Beijing Institute of Biological Products) vaccine.

### 2.3. Sample Collection

Oropharyngeal and nasopharyngeal swabs collected from each patient as part of routine clinical care were placed in a viral transport medium (VTM) tube and transported to the MRC/UVRI and LSHTM Uganda Research Unit’s laboratory in Entebbe for real-time PCR testing and genomic sequencing.

### 2.4. Data Collection 

Data were collected on socio-demographics, previous COVID-19 infection, COVID-19 vaccination status and severity of current infection, and entered by trained study staff onto encrypted computers and tablets into a REDCap database. Vaccination status was captured based on self-reported data but confirmed by crosschecking the participant vaccination cards and on the Uganda Ministry of Health COVID-19 vaccination certification portal [16]. Every patient record was assigned a unique study identification number and access to patient records was limited to only authorized study staff. 

### 2.5. Laboratory Procedures and Molecular Testing

#### 2.5.1. Nucleic Acid Extraction and Real-Time PCR

A volume of 750 μL of VTM eluate was used to extract RNA from nasopharyngeal swab specimens using the Nuclisens EasyMag semi-automated nucleic acid extraction platform (bioMérieux, Marcy-l’Étoile, France) [17]. Real-time PCR was performed using the Berlin [18] and the Life River [19] assay protocols according to the manufacturer’s recommendations and ran on the Quant Studio *7™ Pro* Real-Time PCR System [20].

#### 2.5.2. Deep Sequencing

Genomic sequencing was conducted on all SARS-CoV-2 real-time PCR-positive specimens at the MRC/UVRI & LSHTM Uganda Research Unit’s sequencing platform laboratories in Entebbe. Nasopharyngeal swab specimens that tested positive for SARS-CoV-2 (cycle threshold of <30) were sequenced using an amplicon-based next-generation sequencing (NGS) Illumina COVIDSeq kit [21] on the MiSeq NGS platform [22].

### 2.6. Bioinformatic Analysis

Deep sequence FastQ paired-end reads were assembled using the EDGE Bioinformatics COVID-19 Genome analytics software version 2.4.0 [23] to obtain high-quality SARS-CoV-2 genomes with >85% coverage. Mutation calling was performed using the Next clade software version 3.5.0 [24] and SARS-CoV-2 lineage analysis was conducted using Pangolin version 4.3.1 [25]. Phylogenetic analysis was performed using Iqtree version 2.3.6 [26]. 

## 3. Results 

### 3.1. Population Characteristics 

A total of 1398 participants with a median age of 30 years (interquartile range: 24–41 years) provided nasopharyngeal swabs. The gender distribution was 44.6% males and 55.4% females. Most participants displayed typical COVID-19 symptoms, including fever (>38 °C, 70.4%), cough (89.6%), malaise (68.3%), headache (81.4%), fatigue (68.3%), and others such as shortness of breath (31.1%), loss of smell (37.7%), and chills (40.4%). 

### 3.2. SARS-CoV-2 Lineage Analysis 

Of the 1398 samples tested on real-time PCR, 174 tested positive for SARS-CoV-2 (12.4% positivity rate). Of the SARS-CoV-2 positive samples, 163 were successfully genotyped (94% success rate). All the genotyped sequences were identified as belonging to the Omicron variant. The most prevalent lineages and sublineages were XBB.1* (113 sequences, 69.3%), followed by JN.1* (12 sequences, 7.4%), XBB.2* (11 sequences, 6.7%) and FL* (11 sequences, 6.7%), EG* (7 sequences, 4.3%), with smaller numbers in other categories: 5 sequences (3.1%) classified as ‘others’ and 4 sequences (2.5%) classified as CM* (2.5%) (Table 1). 

**Figure 1:**
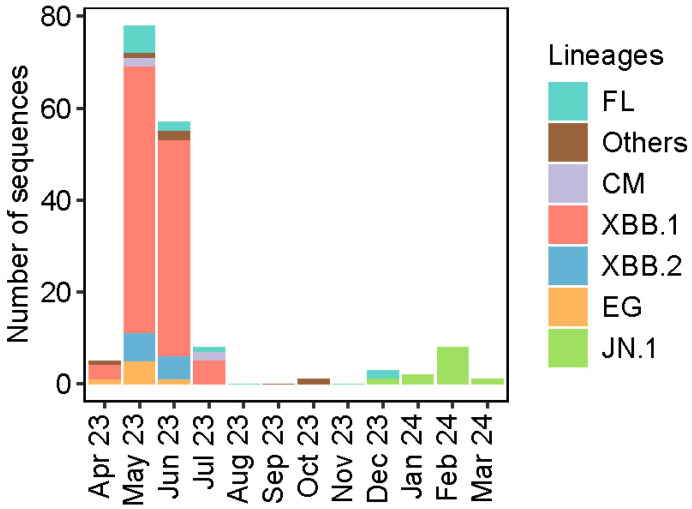
Distribution of SARS-CoV-2 lineages over time (April 2023 to March 2024).

The distribution of SARS-CoV-2 lineages over time showed the dominance of the XBB.1 lineage from April 2023 to July 2023 (Figure 1). After that, there was a general decline in the number of SARS-CoV-2 positive cases at the study sites with almost none sampled between August and September 2023. However, from December 2023, the first JN.1 lineage was detected followed by an increase in the number of JN.1 identified.

**Figure 2:**
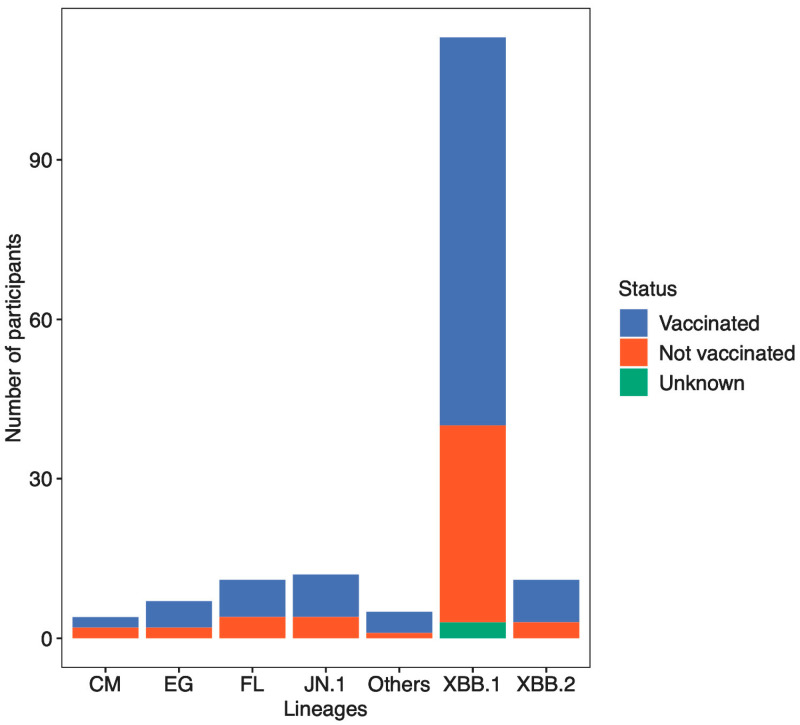
Number of SARS-CoV-2 lineages according to vaccination status. The XBB.1 SARS-CoV-2 lineage generally dominated in both vaccinated and non-vaccinated individuals as well as those whose vaccination status was unknown.

The participants’ (COVID-19 positive individuals) vaccination status was detailed as follows: 107 (65.6%) were vaccinated (received one or more COVID-19 vaccine doses), 53 (32.5%) were not vaccinated and 3 (1.8%) had an unknown vaccination status. Genotyping results showed that 68.2% (73/107) of vaccinated and 69.8% (37/53) of non-vaccinated individuals were assigned the XBB.1 SARS-CoV-2 lineage relative to other detected lineages (Figure 2). The association between vaccination status and lineage assignment was assessed using Fisher’s exact test. There was no statistically significant association between vaccination status and lineage assignment (*p*-value = 0.994). 

### 3.3. Mutation Analysis 

**Figure 3:**
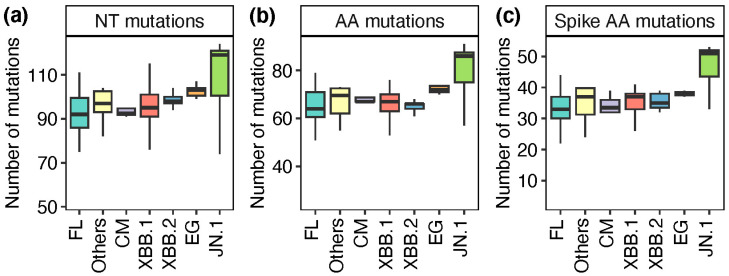
Mutation profile analysis according to SARS-CoV-2 lineages. Plots show the number of nucleotide mutations (panel (**a**)), associated amino acid mutations (panel (**b**)) and spike amino acid mutations (panel (**c**)).

Analysis in Nextclade [24] based on the Wuhan-Hu-1/2019 (MN908947) reference sequence, identified several mutations in the spike protein region (Supplementary Table S1) that included the T19I, A27S, H655Y, N679K, N969K and D614G as the most frequent mutations detected. The distribution of identified nucleotide and amino acid mutations across various lineages is shown in Figure 3. The D614G point mutation has been associated with increased infectivity and replication fitness compared to the original SARS-CoV-2 [27,28]. The JN.1 omicron variant lineage had the highest number of nucleotide mutations compared to any other variant identified in this study with spike protein mutations that included the R21T, S50L, V127F, R158G as well as the L455S. The JN.1 variant’s increased transmissibility and immune evasion potential have been attributed to these mutations [6,29]. Notably, a distinctive L455S mutation in the spike protein sets JN.1 apart from its parent lineage, BA.2.86 [30].

**Figure 4:**
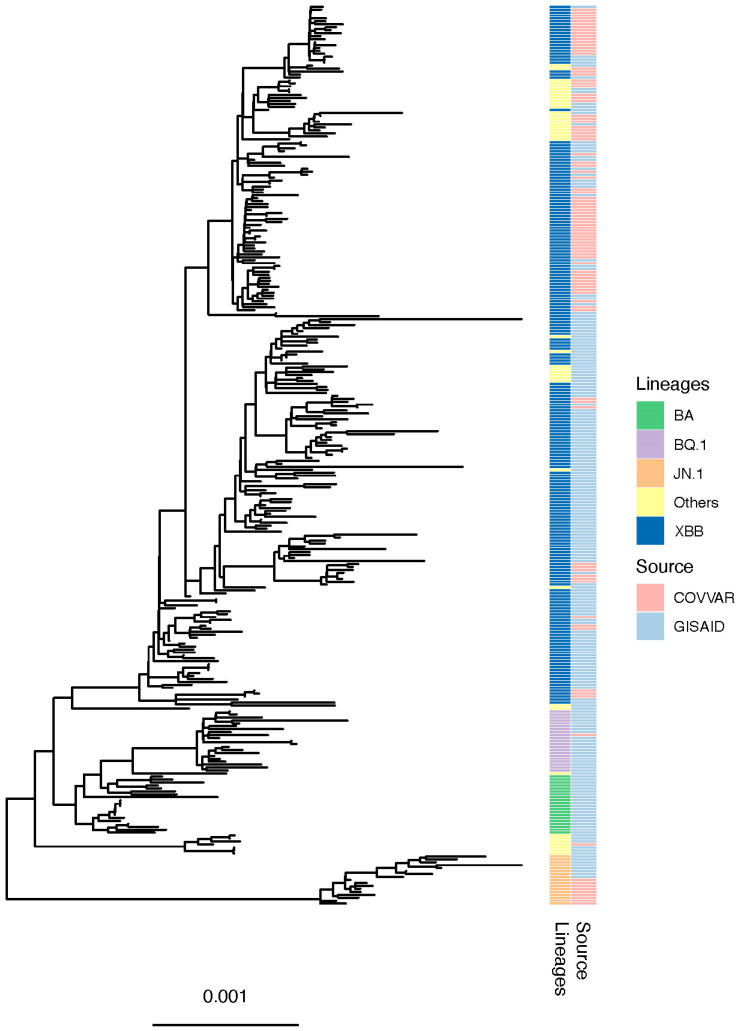
Maximum likelihood phylogenetic tree (1000 bootstraps). Includes 163 SARS-CoV-2 sequences from the COVVAR study and 206 Ugandan sequences from the GISAID database.

We retrieved 206 Ugandan sequences from GISAID (spanning a period of 2023–2024) to supplement those generated from the COVVAR study. A multiple sequence alignment was generated using MAFFT [31]. We reconstructed a maximum likelihood phylogenetic tree (Figure 4) using IQ-TREE [26] based on a GTR+F+R2 model of nucleotide substitution chosen as the optimum model using the Jmodel test [32]. The phylogenetic tree was visualized using phytools [33] and ggtreeExtra packages in R [34]. Phylogenetic analysis showed that XBB* SARS-CoV-2 lineages and sublineages constituted the majority of Ugandan sequences generated from both the COVVAR study and GISAID database. However, the JN.1 lineage formed a separate monophyletic cluster on the phylogenetic tree for sequences generated from the COVVAR study and those deposited in GISAID, highlighting its substantial genetic and evolutionary divergence relative to other SARS-CoV-2 lineages that could be attributed to its notable spike protein mutation profile.

## 4. Discussion

In our study, we identified the Omicron XBB.1 lineage and its sub-lineages as the most dominant, which is consistent with the findings of other studies carried out globally in 2023 [35,36,37]. This dominance can be attributed to the heightened immune evasion capabilities acquired by variants within this lineage [37]. The XBB lineage, identified in September 2022, originated through the recombination of two BA.2-derived variants, gradually supplanting pre-existing Omicron strains. Characterized by heightened transmissibility rates and immune evasion properties, members of the XBB lineage have been responsible for initiating small waves of infections across numerous countries, albeit with a heterogeneous geographical distribution. For instance, the subvariant XBB.1.5 colloquially referred to as “Kraken” has been the prevailing strain for the majority of 2023 [38].

Notably, the emergence of the JN.1 sub-lineage has rapidly replaced other strains to become predominant in multiple countries, demonstrating its competitive advantage [39]. The presence of other subvariants, such as XBB.2, EG.5.2, and JN.1, detected at smaller proportions, underscores the genetic diversity within the Omicron lineage. While these variants were present at low frequencies, their detection emphasizes the importance of continued genomic surveillance to monitor the emergence and circulation of novel variants [38,40]. Understanding the prevalence and distribution of these lineages and sub-lineages could inform public health strategies, such as vaccine development and surveillance efforts [41,42]. It was also noteworthy that a small proportion of samples exhibited variants such as the EG.5—also known as ‘Eris’, a variant that emerged around February 2023 and rapidly spread in over 50 countries—and BA.2.86, the ancestral virus of the JN.1. The BA.2.86 has about 60 more spike protein mutations than the original or parent virus and over 30 more mutations than its close Omicron relatives, the BA.2 and earlier dominant XBB.1.5 variant. 

The JN.1 spread rapidly to become the most widely circulating lineage in the United States and accounted for more than 80% of all circulating variants by the end of January 2024 [43]. It has established itself as the dominant global strain [44]. To date, only 18 JN.1* SARS-CoV-2 Omicron sub-variant cases have been reported in Uganda in GISAID [45]. Several VoC SARS-CoV-2 mutations that included E484K and P681R were initially identified in the Alpha and Beta variants in early 2021. It is unknown why those mutations were not detected in the Omicron family of viruses but returned in the JN.1 Omicron lineage. In Uganda, more extensive genomic surveillance is needed to assess the extent of spread of JN.1 as well as monitor other SARS-CoV-2 variants that are currently circulating to inform prevention efforts. Recent genotyping of community samples in Uganda conducted between May and June 2024 has shown that over 90% of identified lineages and sub-lineages belong to the JN.1* of SARS-CoV-2 (unpublished data). This highlights the virus’ fitness to outcompete previously dominant lineages. Genomic sequencing plays a key role in monitoring the evolution of the SARS-CoV-2 genome and provides information on the genetic characteristics of recently circulating or dominant SARS-CoV-2 strains that is critical for COVID-19 vaccine design research. 

## 5. Conclusions

Overall, genotypic data showed that the Omicron variant, particularly the XBB.1 lineage, was predominant until July 2023. Since then, other lineages and sub-lineages of the JN.1 variant have been increasingly detected and designated as variants of interest as of 3 May 2024 [46]. Our results contribute to an understanding of the genetic diversity and distribution of Omicron subvariants within the analyzed population. Further comparative analysis with additional datasets and ongoing genomic surveillance efforts will be crucial for tracking the evolution and spread of SARS-CoV-2 variants, informing effective public health responses. As JN.1 continues to evolve and is poised to replace the XBB.1 sublineage, ongoing surveillance, vaccination strategies, and adherence to preventive measures are crucial to mitigating its potential impact on global public health. A limitation of this study was the small number of positive samples as a result of the declining number of SARS-CoV-2 positive cases at the study sites, particularly after July 2023. However, despite the small sample size, the study identified important trends in the evolution of SARS-CoV-2 variants in Uganda including the appearance and increased prevalence of the JN.1 variant. 

## Figures and Tables

**Table 1:** Number and Proportion of SARS-CoV-2 Lineage assignment and vaccine status.

Lineage	Sub Lineages (*n*)	All (*n* = 163)	Vaccinated (*n* = 107)	Unvaccinated (*n* = 53)	Unknown Vaccination Status (*n* = 3)
		*n* (%)	*n* (%)	*n* (%)	*n* (%)
CM	CM.8.1 (4)	4 (2.5)	2 (1.9)	2 (3.8)	-
EG	EG.2 (1), EG.5.2 (6)	7 (4.3)	5 (4.7)	2 (3.8)	-
FL	FL.1.5.1(1), FL.10(4), FL.10.2 (1), FL.13.4.1 (1), FL.2.2 (4)	11 (6.7)	7 (6.5)	4 (7.5)	-
JN.1	JN.1 (10), JN.1.18 (1), JN.1.4.7 (1)	12 (7.4)	8 (7.5)	4(7.5)	-
Others	BQ.1.1 (1), FY.4.1 (1), FY.4.1.2 (1), GY.2.1 (1), HK.27.1 (1)	5 (3.1)	4 (3.7)	1 (1.9)	-
XBB.1	XBB.1 (2), XBB.1.16 (21), XBB.1.16.1 (6), XBB.1.34.1 (14), XBB.1.5 (54), XBB.1.5.12 (2), XBB.1.5.28 (2), XBB.1.5.63 (2), XBB.1.9.1 (10)	113 (69.3)	73 (68.2)	37 (69.8)	3 (100)
XBB.2	XBB.2.3 (6), XBB.2.3.11 (2), XBB.2.3.4 (3)	11 (6.7)	8 (7.5)	3 (5.7)	-

*n*, number; %, column percentage.

## Data Availability

All SARS-CoV-2 nucleotide sequence data are available and have been deposited in the GISAID database and is publicly available with the following accession numbers: EPI_ISL_19139431-19139566, EPI_ISL_19139747-19139765 and EPI_ISL_19164604-19164611.

## Supplementary Materials

The following supporting information can be downloaded at: https://www.mdpi.com/article/10.3390/v16121860/s1. Table S1: Detailed nucleotide and amino acid mutation list.
